# Clinical outcomes of muscle invasive bladder Cancer according to the BASQ classification

**DOI:** 10.1186/s12885-019-6042-1

**Published:** 2019-09-09

**Authors:** Hyeong Dong Yuk, Chang Wook Jeong, Cheol Kwak, Hyeon Hoe Kim, Kyung Chul Moon, Ja Hyeon Ku

**Affiliations:** 1 Department of Urology, Inje University College of Medicine, Inje University Sanggye Paik Hospital, Seoul, Korea; 2 Department of Urology, Seoul National Univervity College of Medicine, Seoul National University Hospital, Seoul, Korea; 3 Department of Pathology, Seoul National Univervity College of Medicine, Seoul National University Hospital, Seoul, Korea

**Keywords:** Basal cell, Immunohistochemistry, Molecular subtype, Neoplasm metastasis, Squamous cell, Urinary bladder neoplasms

## Abstract

**Background:**

We evaluated the clinical efficacy and prognosis of muscle-invasive bladder cancer according to the basal/squamous-like (BASQ) classification system based on immunohistochemical staining [CK5/6(+), CK14(+), GATA3(−), and FOXA1(−)].

**Methods:**

One hundred patients diagnosed with muscle-invasive bladder cancer (cT2-4 N0-3 M0) were included in the study. All patients underwent radical cystectomy after transurethral removal of bladder tumor. Immunostaining was performed for CK5/6, CK14, FOXA1, and GATA3 antibodies on tissue microarray slides, and expression patterns were quantitatively analyzed using a scanning program.

**Results:**

The median follow-up time was 77.4 (interquartile range: 39–120.9) months. The mean age of the patients was 65.1 ± 11.2 years. FOXA1 or CK14 expression greater than 1% was respectively positively and negatively correlated with overall survival (OS; *p* = 0.011 and *p* = 0.042, respectively), cancer-specific survival (CSS; *p* = 0.050 for both), and recurrence-free survival (RFS; *p* = 0.018 and *p* = 0.040, respectively). For CK5/6+ and GATA3- or FOXA1- expression, 10% CK5/6+ cells were negatively correlated with OS (*p* = 0.032 and *p* = 0.039, respectively) and with RFS in combination with FOXA1- only (p = 0.050).

**Conclusions:**

In this study, CK14 expression was associated with a poor prognosis. The new classification system of bladder cancer based on molecular characteristics is expected to helpful tool for the establishment of personalized treatment strategies and associated prediction of therapeutic responses.

**Electronic supplementary material:**

The online version of this article (10.1186/s12885-019-6042-1) contains supplementary material, which is available to authorized users.

## Background

Bladder cancer is the fourth most common cancer in men, with approximately 60,000 new diagnoses each year [1], ranking as the eighth leading cause of cancer-related deaths in the United States, with about 12,000 deaths annually [1]. Specifically, in 2017, there were 79,030 cases of bladder cancer and 16,870 related deaths in the United States [1]. Approximately 90–95% of all bladder cancer cases are urothelial cell carcinoma, with the minority consisting of non-urothelial cell carcinoma. During initial diagnosis, 70–80% of bladder cancers are diagnosed as non-invasive with the remaining 20–30% diagnosed as invasive. Most cases of non-invasive bladder cancer can be treated with transurethral removal of the bladder tumor (TURB) alone [2, 3]. However, a high recurrence rate after TURB has been reported within 1 year (15–70%) and 5 years (7–40%) [2, 3]. Therefore, continuous additional testing and repeated treatments are often needed. Indeed, in the United States, bladder cancer is reportedly one of the tumors for which patients incur a high costof [2, 3].

Recently, a large-scale, detailed analysis of the molecular genetic characteristics of bladder cancer was reported through The Cancer Genome Atlas (TCGA) [2, 3]. The TCGA study revealed that bladder cancer can be classified into several subtypes depending on the molecular characteristics of the genomes [4–9]: luminal type, basal type, p53-like tumor, and small cell carcinoma-like tumor. Among these subtypes, the basal type is associated with a particularly poor prognosis [4–9]. Moreover, the basal type and p53-like tumor are highly resistant to preoperative chemotherapy; thus, identifying the accurate subtype is an essential factor in clinical decision-making [5]. Basal/squamous-like (BASQ) is a basal type of bladder cancer with a very poor prognosis and high rate of resistance to chemotherapy [5]. It is immunohistochemically defined by CK5/6(+), CK14(+), GATA3(−) and FOXA1(−) expression [5]. However, there is no report on the treatment response and prognosis of patients with bladder cancer when applying this new classification system. Therefore, in the present study, we evaluated the clinical efficacy and prognosis of MIBC according to the use of the BASQ classification system in clinical practice.

## Methods

### Ethics

This study was approved by the Institutional Review Board (IRB No. H-1806-081-951). We used the human bladder cancer materials stored in the cancer tissue bank (IRB No. H1307–084-505). We obtained informed consent from all research participants.

### Patient populations

A total of 100 patients with muscle-invasive urothelial carcinoma (cT2-4 N0-3 M0) of the urinary bladder were included in the study. Patient selection was based on the availability of sufficient material for immunohistochemistry. All patients underwent TURB followed by radical cystectomy between 2000 and 2012 at Seoul National University Hospital.

### Tissue microarray (TMA) construction

Hematoxylin and eosin slides were reviewed for confirmation of the pathologic diagnosis and various pathologic parameters, including invasion depth and grade. We constructed TMA blocks from formalin-fixed paraffin-embedded tissue blocks (Superbiochips Laboratories, Seoul, Korea). In brief, two representative tumor cores (2 mm in diameter) were selected from the viable tumor area. The cancer tissues of patients were examined microscopically by a skilled pathologist, and the TMA was prepared after selecting the most representative cancer tissues. Immunostaining was performed for CK5/6, CK14, FOXA1, and GATA3 antibodies on TMA slides from the 100 patient samples, and the expression patterns were quantitatively analyzed using a scanning program. Based on the expression patterns, the patients were divided according to the BASQ classification (CK5/6, CK14, FOXA1, and GATA3).

The prognostic value of the BASQ classification was determined based on clinical and pathological information such as age, body mass index, sex, American Society of Anesthesiologists (ASA) physical status, pathologic TNM stage, carcinoma in situ status, lymphovascular invasion, margin-positive status, lymph node dissection range, number of removed lymph nodes, number of positive lymph nodes, and neoadjuvant chemotherapy enforcement. We also collected various types of oncological data, including the recurrence, mortality, and cancer-related mortality rates.

### Immunohistochemistry (IHC)

IHC staining was performed on 4-μm-thick sections from TMA blocks using the Benchmark XT autostainer (Ventana Medical Systems, Tucson, AZ, USA). The sections were incubated with the following primary antibodies: mouse monoclonal antibodies against CK5/6 (64 min; 1:50; Dako, Glostrup, Denmark), CK14 (32 min; 1:50; Cell Marque, Rocklin, CA, USA), and GATA3 (32 min; 1:500; clone 156-3C11; Cell Marque), and rabbit polyclonal antibody against FOXA1 (16 min; 1:700; ThermoFisher Scientific, Rockford, IL, USA). To interpret the IHC results, the percentage of positively stained tumor cells was semi-quantitatively evaluated into three categories; 0, no positive cells; 1+, 1–10% positive cells; 2+, 11–25% positive cells; 3+, > 25% positive cells.

### Statistical analysis

Continuous variables are presented as the median value and interquartile ranges (IQRs) or average value and standard deviations (SDs). Nominal variables are presented as the frequency of events (%). The primary endpoint of the study was the overall survival (OS) rate, and the secondary endpoints were cancer-specific survival (CSS) and recurrence-free survival (RFS). The Kaplan-Meier method was used to predict all survival outcomes, and significance among groups was determined using log-rank tests. Cox proportional hazards regression analysis was used for analysis of various oncology outcomes and predictors. All statistical tests were performed using IBM SPSS Statistics version 22.0 (IBM, Armonk, NY, USA) and STATA version 14 (StataCorp LP, College Station, Texas). A *p*-value < 0.05 was considered statistically significant.

## Results

### Baseline characteristics of the patients

Table 1 shows the basic characteristics of the 100 patients involved in the study. The median follow-up time was 77.4 (IQR: 39–120.9) months. The mean age of the patients was 65.1 ± 11.2 years, and more than 80% of the patients were males. Ninety-one patients (91%) had an ASA physical status below 3. All patients were diagnosed as having muscle-invasive bladder cancer with T2-4 N0-3 M0; 10% of the patients underwent neoadjuvant chemotherapy, 35% of the patients underwent radical cystectomy with standard pelvic lymph node dissection (PLND), whereas 65% of the patients had extended PLND. Moreover, 65% of the patients underwent ileal conduit urinary diversion, and the remaining 35% underwent neobladder diversion.

**Table 1:** Basic patient characteristics

Variable	*N* = 100
Age (mean ± SD) (year)	65.1 ± 11.2
BMI (m2/kg)	23.56 ± 5.93
Gender	
Female	17 (17.0%)
Male	83 (83.0%)
ASA	
1	34 (34.0%)
2	57 (57.0%)
≥ 3	9 (9.0%)
pT stage	
T2	50 (50.0%)
T3	42 (42.0%)
T4	8 (8.0%)
LVI	42 (42.0%)
CIS	34 (34.0%)
N stage	
N0	80 (80.0%)
N1	7 (7.0%)
N2	8 (8.0%)
N3	5 (5.0%)
LND range	
Standard	36 (36.0%)
Extend	64 (64.0%)
Removed LN	14.1 ± 12.9
Positive LN	1.0 ± 2.8
Recurrence	31 (31.0%)
Mortality	59 (59.0%)
Cancer related mortality	32 (32.0%)

*BMI* Body mass index, *LVI* Lymphovascular invasion, *CIS* Carcinoma in situ, *LND* Lymph node dissection, *LN* Lymph node;

### Prognostic significance of FOXA1, GATA3, CK14, and CK5/6 expression

**Fig. 1:**
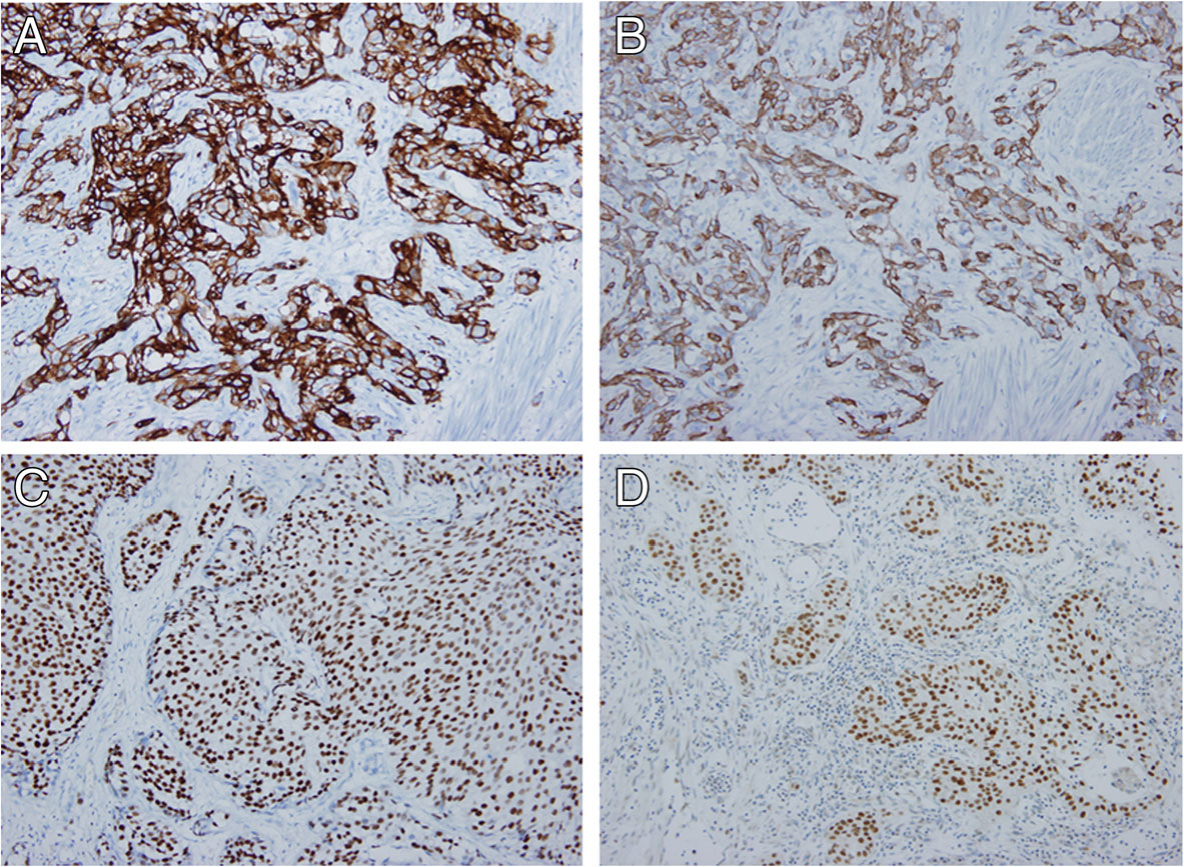
Positive immunohistochemical staining of CK5/6 (**a**), CK14 (**b**), GATA3 (**c**), and FOXA1 (**d**). CK5/6, CK14 showed membranous staining, and GATA3, FOXA1 revealed nuclear positivity

**Fig. 2:**
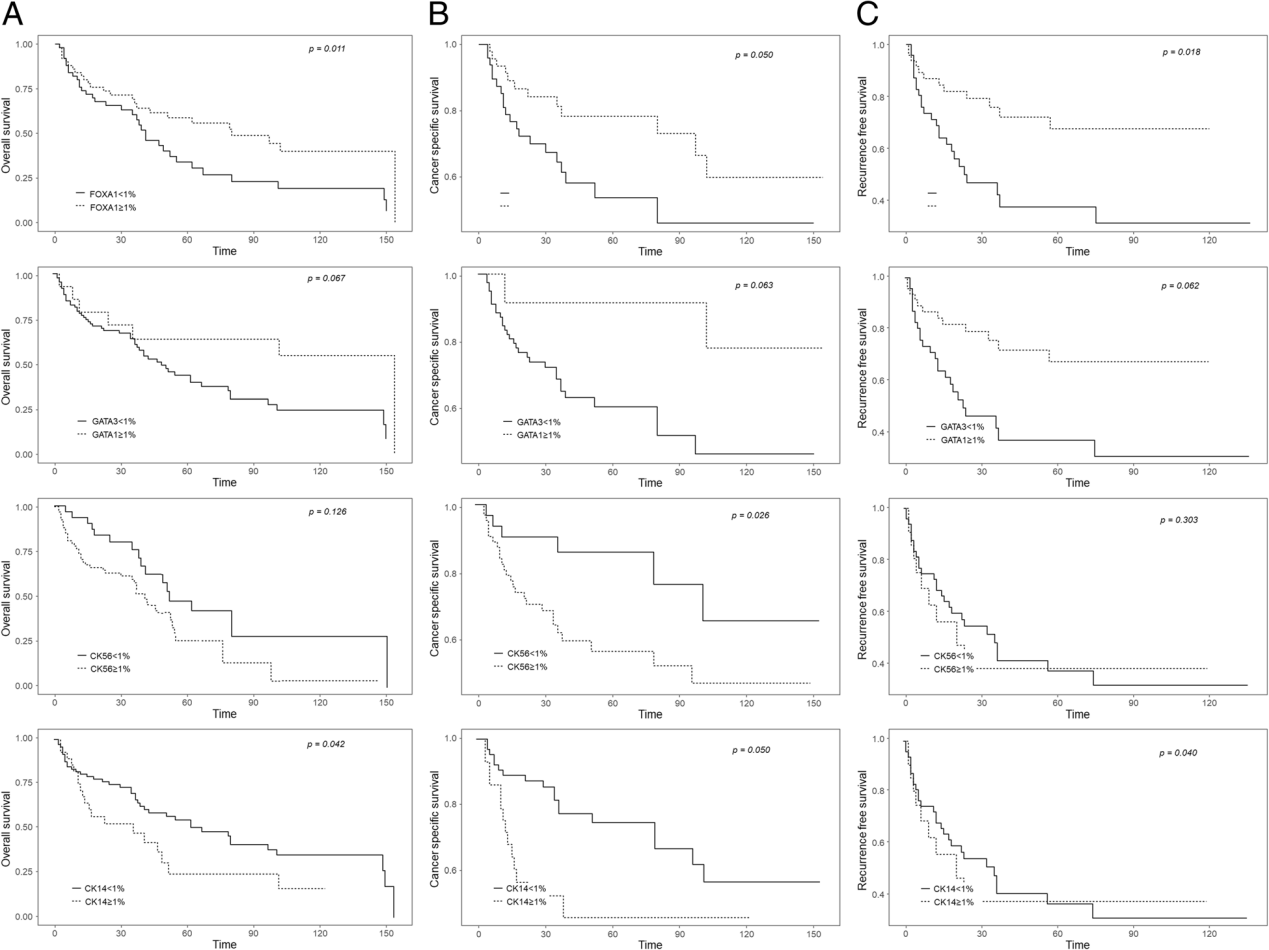
Oncologic outcomes according to subtypes of urothelial carcinoma. **a** overall survival, **b** cancer specific survival, **c** recurrence free survival

Table 2 shows semi-quantitatively evaluated IHC results. CK5/6 and CK14 staining showed membranous expression, and GATA3 and FOXA1 staining was present in the nucleus (Fig. 1). A frequency of FOXA1 expression greater than 1% was positively correlated with OS (*p* = 0.011), CSS (*p* = 0.050), and RFS (*p* = 0.018) (Fig. 2). In addition, a FOXA1 positive frequency greater than 10% was positively correlated with CSS (*p* = 0.022), and a frequency above 25% was positively correlated with RFS (p = 0.011).

**Table 2:** Multivariable Cox regression analysis of overall survival, cancer specific survival, recurrence free survival

Parameter	Overall survival		Cancer specific survival		Recurrence free survival	
	HR (95% CI)	*P*-value	HR (95% CI)	*P*-value	HR (95% CI)	*P*-value
pT stage						
T2	reference		reference		Reference	
≥ T3	1.46 (1.15–1.85)	0.002	1.49 (1.17–1.90)	0.001	2.04 (0.38–10.74)	0.400
LVI	0.86 (0.25–2.93)	0.806	2.33 (0.44–12.16)	0.316	2.66 (0.97–7.65)	0.060
CIS	0.90 (0.28–2.89)	0.858	4.15 (1.21–16.03))	0.028	0.70 (0.43–1.14)	0.160
N stage		0.478		0.096		0.096
N0	reference		Reference		reference	
≥ N1	1.71 (1.46–1.99)	< 0.001	1.75 (1.62–1.91)	0.004	2.96 (1.40–6.22)	0.004
CK5/6	3.30 (0.81–14.63)	0.101	2.57 (0.90–8.16)	0.088	3.15 (0.8–12.63)	0.104
CK14	6.16 (1.28–38.30)	0.033	3.96 (1.13–16.36)	0.040	3.19 (1.07–9.55)	0.037
GATA3	0.77 (0.37–1.58)	0.477	0.81 (0.22–2.89)	0.742	0.29 (0.01–5.27)	0.409
FOXA1	0.08 (0.01–0.59)	0.023	0.08 (0.01–0.61)	0.024	0.12 (0.1–1.51)	0.103

*HR* Hazard ratio, *CI* Confidence interval, *LVI* Lymphovascular invasion, *CIS* Carcinoma in situ, *LND* Lymph node dissection, *LN* Lymph node, *UC* Urothelial carcinoma

OS, CSS, and RFS all tended to improve in patients with ≥1% GATA3 expression compared to those with < 1% expression, although the difference was not statistically significant (Fig. 2). GATA3 expression greater than 10% was positively correlated with RFS (*p* = 0.032).

A CK14 expression rate greater than 1% was negatively correlated with OS (*p* = 0.042), CSS (*p* = 0.050), and RFS (*p* = 0.040) (Fig. 2). Similarly, OS and RFS tended to be worse in patients with ≥1% CK5/6 expression than in patients with < 1% CK5/6 expression but not significantly (Fig. 2). However, CSS was better in patients with < 1% CK5/6 expression than in those with ≥1% expression (*p* = 0.028).

Additional file 1 shows nomogram for predictors of survival after cystectomy. In multivariable Cox regression analysis, OS was significantly correlated with the expression of CK14 (HR: 6.16, 95% CI: 1.28–38.30) and FOXA1 (HR: 0.08, 95% CI: 0.01–0.59) in the urothelial carcinoma subtype (Table 3). In CSS, expression of CK14 (HR: 3.96, 95% CI: 1.13–16.36) and FOXA1 (HR: 0.08, 95% CI: 0.01–0.61) was also significantly correlated. CK14 was negatively correlated with OS and CSS, and FOXA1 was positively correlated with OS and CSS (Table 3). In RFS, only CK14 was negatively correlated with RFS (HR: 3.19, 95% CI: 1.07–9.55).

**Table 3:** Immunohistochemistry results

	CK14	CK5/6	GATA3	FOXA1
< 1%	60 (60.0%)	24 (24.0%)	8 (8.0%)	15 (15.0%)
1~10%	12 (12.0%)	35 (35.0%)	11 (11.0%)	15 (15.0%)
11~25%	15 (15.0%)	16 (16.0%)	14 (14.0%)	20 (20.0%)
> 25%	13 (13.0%)	25 (25.0%)	67 (67.0%)	50 (50.0%)

**Fig. 3:**
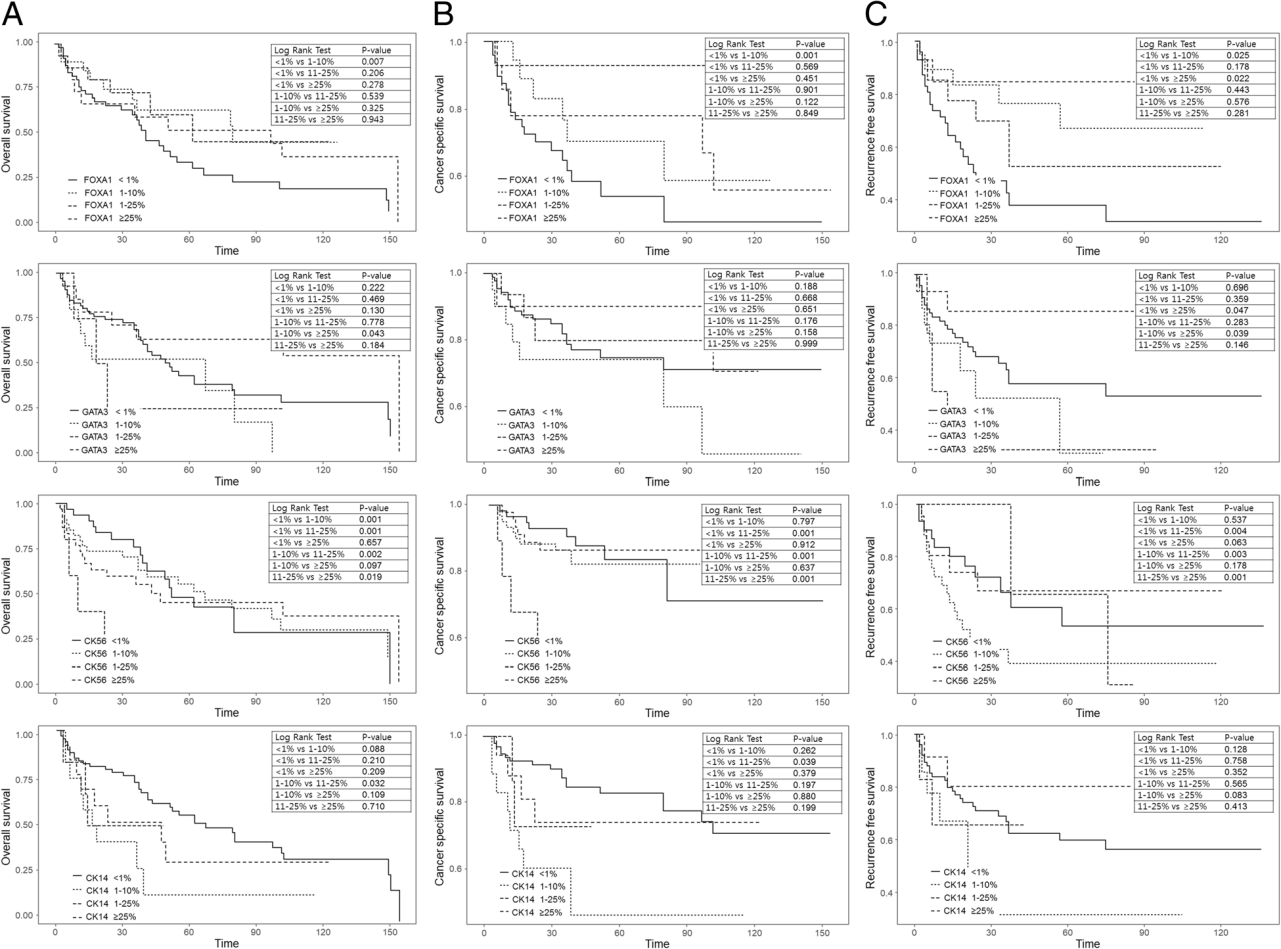
Comparison of oncologic outcome according to expression level of subtypes of urothelial carcinoma. **a** Overall survival, **b** Cancer specific survival, **c** Recurrence free survival

A comparison of oncologic outcomes between the < 1%, 1–10%, 11–25, > 25% groups showed that FOXA1 expression in the 1–10% group was positively correlated with OS compared to that in less than 1%; OS (*p* = 0.007), CSS (*p* = 0.001), and RFS group (*p* = 0.025) (Fig. 3). CK14 was negatively correlated with OS, CSS, and RFS according to subtype expression level. A comparison of oncologic outcomes showed that in both the lesser than 1% and between 11 and 25% groups, CK14 expression between 11 and 25%, was negatively correlated with OS compared to that in lesser than 1%; OS (p = 0.001), CSS (p = 0.001), and RFS (*p* = 0.004) (Fig. 3). A comparison of oncologic outcomes between the 1 and 10% and between 11 and 25% groups showed that CK14 expression in the between 11 and 25% group was negatively correlated with OS compared to that in the lesser than 1%; OS (*p* = 0.002), CSS (p = 0.001), and RFS group (*p* = 0.003) (Fig. 3).

### Relationship between basal type and prognosis

**Fig. 4:**
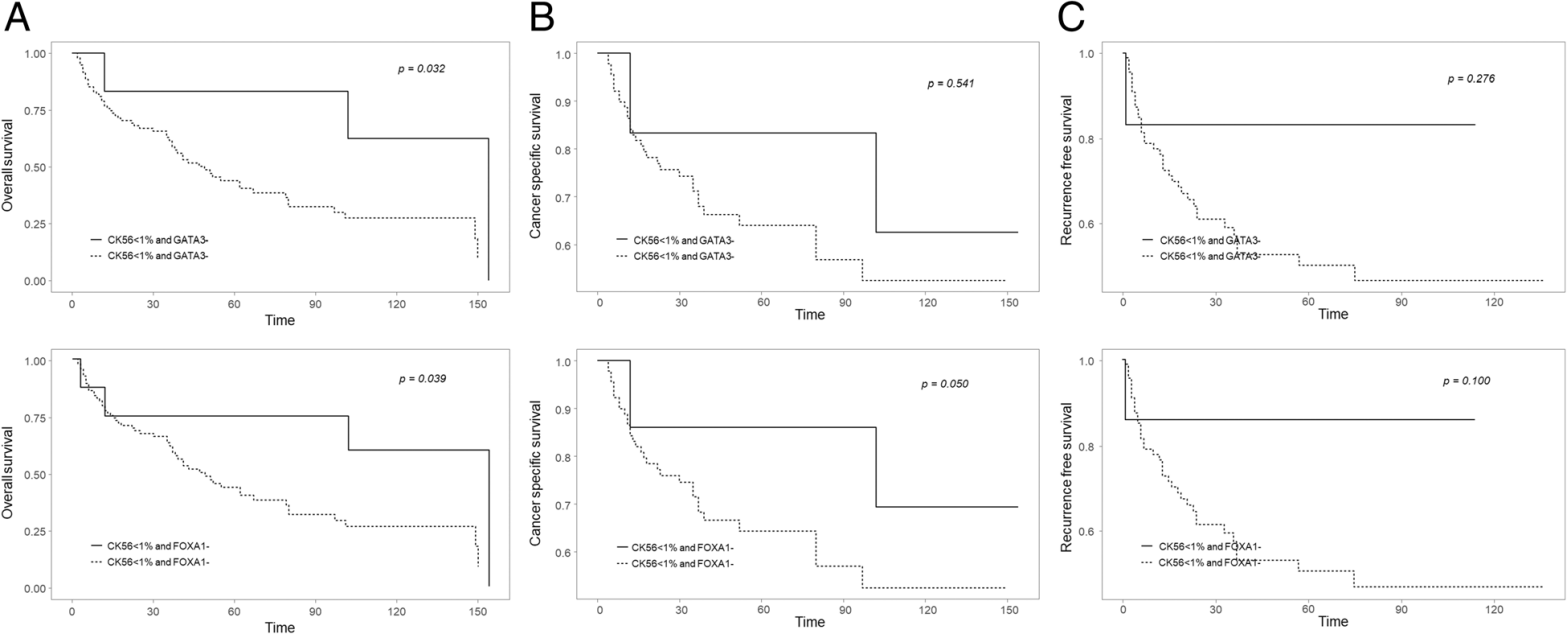
Oncologic outcomes according to ck5/6(+) and gata3(−) and ck5/6(+) and foxa1(−) in immunochemical staining. **a** Overall survival, **b** Cancer specific survival, **c** Recurrence free survival

In the case of CK5/6+ and GATA3- samples, more than 1% CK5/6 expression and GATA3- expression was significantly negatively correlated with OS (*p* = 0.032; Fig. 4). In the case of CK5/6+ and FOXA1- samples, more than 1% CK5/6+ expression and FOXA1 expression was significantly negatively correlated with OS and CSS (*p* = 0.039 and *p* = 0.050, respectively; Fig. 4). In the case of CK14+ and GATA3-samples and CK14+ and FOXA1- samples were not significantly correlated with OS, CSS and RFS.

## Discussion

Several recent studies have shown that in addition to the well-known Clinic factors, various antropometric factors have an effect on the outcome of the bladder cancer [10–13]. The recurrence rate of bladder cancer is reported to be significantly higher in obese patients than in normal weight patients [10, 13]. Metabolic features such as obesity and associated insulin resistance have also been reported to affect prognosis [13]. High BMI also helps to predict poor prognosis, such as lymph node metastasis [10]. Immunological and inflammation markers such as basophil count, neutrophil and lymphocyte count, and C-reactive protein are also helpful in predicting recurrence after cystectomy or intravesical Bacillus Calmette-Guérin (BCG) treatment [11, 12].

In addition to these various antropometric factors, histologic features have been reported to be helpful in predicting the prognosis of bladder cancer. Recent molecular studies have provided new insight into the factors contributing to bladder cancer development and progression. TMAs have been used to analyze genome expression, and immunohistochemical expression patterns are used to classify unique molecular types of bladder cancer. The gene mutations identified to date include genes related to chromatin regulation, cell cycle regulation, and kinase signaling pathways. In particular, molecular insight has been gained with respect to the cell and molecular biology of the urothelium, with 32 gene mutations significantly and repeatedly observed in urothelial cell carcinoma, including genes related to cell cycle regulation, chromatin regulation, and kinase signaling pathways [5]. In particular, tumor protein 53 (TP53), fibroblast growth factor receptor-3 (FGFR3) mutations, and genes involved in the phosphatidylinositol-3-OH kinase (Pl3K)/protein kinase B (Akt)/mammalian target of rapamycin (mTOR) pathway were found to be associated with the prognosis of bladder cancer [5].

Besides the specific mutations and pathways, the discovery of molecular subtypes of urothelial cell carcinoma represents another important advance obtained through molecular studies. Several studies on genome expression profiles have reported that bladder cancer can be categorized into two intrinsic molecular types: luminal and basal, which are similar to those in breast cancer [5, 14, 15]. The molecular subtype of urothelial carcinoma is related to cell differentiation [16]. Basal type and luminal type are distinguished by keratin markers. The basal type has keratins representing the basal/stem-cell compartment, and the luminal type has keratins representing the umbrella cell layer [6, 15]. Basal type keratins are associated with the transcription factor ΔNP63, which is related to a poor prognosis of muscle-invasive bladder cancer [15, 16].

Lindgren et al. [8] first classified samples from 144 patients with urothelial cell carcinoma according to gene expression patterns. They divided the urobasal group into two subgroups: urobasal A and B, according to their molecular characteristics. Urobasal A was mostly a non-muscle invasive bladder cancer; however, patients with urobasal B showed a progressive phenotype with increased cell cycle activity and basal cell-related keratin expression [7].

The MD Anderson cohort was classified into basal and luminal types, which included 98 patients with invasive bladder cancers and 34 patients with superficial bladder cancers. The luminal type showed strong expression of markers such as CD24, FOXA1, GATA3, CK20, and XBP1, whereas the basal type was characterized by high-molecular-weight keratins (CK5 and CK14) and strong expression of CDH3 and CD44 [5, 17].

Thus, the molecular characteristics of urothelial carcinoma can be used to predict the therapeutic effect and prognosis of the patient. McConkey et al.[18] reported that these molecular characteristics could predict the benefits of treatment such as chemotherapy or target agent therapy. Specifically, basal subtypes have been shown to be beneficial in neoadjuvant settings.[18].

Our present study also showed a tendency for a better prognosis in cancers with FOXA1 or GATA3 expression. Conversely, some of the CK14 and CK5/6-positive cases showed a tendency to be correlated with a poor prognosis. CK 14 negatively correlated with OS, CSS, and RFS, and FOXA1 positively correlated with OS and CSS. The expression of CK14 and FOXA1 subtypes seemed to be correlated with oncologic outcomes compared to those of CK56 and GATA3. Indeed, CK14 and FOXA1 expression may be a sensitive criterion for further differentiating urothelial carcinoma. However, our study was limited to 100 subjects and the results may be due to these limited subjects.

The difference in survival outcomes according to the degree of subtype expression was not significantly correlated with oncologic outcomes. However, CK14 and FOXA1 expression was correlated with oncologic outcomes at some yields. FOXA1 expression in the between 1 and 10% group was positively correlated with OS compared to that in the lesser than 1% group, OS (*p* = 0.007), CSS (*p* = 0.001), and RFS (*p* = 0.025) (Fig. 3).

CK14 also showed differences in oncologic outcome of OS and CSS according to subtype expression levels. There was a difference in the oncologic outcomes between the less than 1%, between 11 and 25%, and more than 25% groups. CK14 is negatively correlated with OS, CSS, and RFS according to subtype expression level (Fig. 3).

Even if FOXA1 is statistically significant in multivariate Cox regression analysis, the odd ratio is 0.08 and its impact is unclear. However, CK14 expression was associated with oncologic outcome of OS and CSS. (Table 3).

In the case of basal type cancers (CK14+, CK5/6+, FOXA1-, GATA3-), CK5/6+ and GATA3- were significantly correlated with a poor OS when the CK5/6+ expression rate was > 10%. CK5/6+ and FOXA1- were also significantly correlated with a poor OS and RFS when the CK5/6+ expression rate was > 10 and > 25%. When we defined the basal type according to the new consensus, we found a significant correlation with poor OS, and a tendency toward an association with RFS. This somewhat unclear correlation is likely due to the insufficient number of specimens analyzed in our study. However, this finding suggests a clear relationship between the basal type and a poor prognosis.

This study has some limitations. This study had a retrospective design, and the sample size was relatively small. Therefore, more extensive and prospective studies are needed to verify the observed associations. And we did not consider the number of TURBs or intravesical treatments that could affect the outcome. Nevertheless, it is meaningful that this study applied the newly established BASQ classification to the evaluation of clinical specimens from patients diagnosed with bladder cancer and related the BASQ classification to prognosis. We could also confirm that the basal and luminal types in the BASQ classification are closely related to patient prognosis.

## Conclusions

In this study, CK14 expression was associated with a poor prognosis. The new classification system of bladder cancer based on molecular characteristics is expected to helpful tool for the establishment of personalized treatment strategies and associated prediction of therapeutic responses.

## Additional file

Additional file 1: **Figure S1.** Nomogram for prediction of survival after cystectomy. (TIF 21 kb)

## Data Availability

The datasets used and/or analysed during the current study are available from the corresponding author on reasonable request.

## Abbreviations

Akt: protein kinase B
ASA: American society of anesthesiologists
BASQ: basal/squamous-like
FGFR3: fibroblast growth factor receptor-3
IHC: Immunohistochemistry
mTOR: mammalian target of rapamycin
Pl3K: phosphatidylinositol-3-OH kinase
PLND: pelvic lymph node dissection
TCGA: the cancer genome atlas
TMA: tissue microarray
TP53: tumor protein 53
TURB: transurethral removal of the bladder tumor
