# Supplementary figures and images for: Clinical outcomes of muscle invasive bladder Cancer according to the BASQ classification
Source: BMC Cancer. 2019 Sep 9;19:897. doi: 10.1186/s12885-019-6042-1 (PMC6734465; doi:10.1186/s12885-019-6042-1)

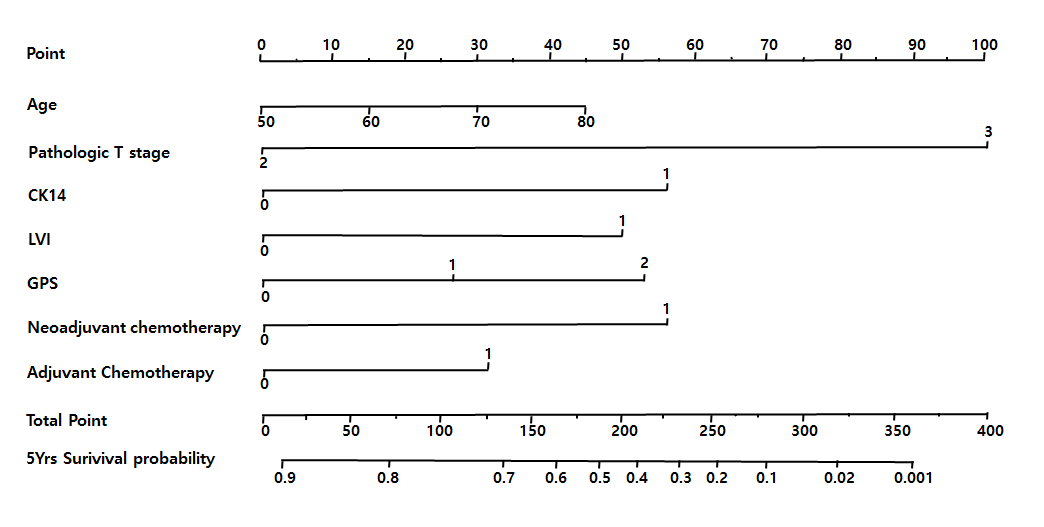

Supplement: Supplementary file 1 — Figure S1. Nomogram for prediction of survival after cystectomy. (TIF 21 kb) [file 12885_2019_6042_MOESM1_ESM.tif]
